# Feasibility of improving vocal fold pathology image classification with synthetic images generated by DDPM-based GenAI: a pilot study

**DOI:** 10.1007/s00405-025-09443-4

**Published:** 2025-05-17

**Authors:** Iman Khazrak, Shahryar Zainaee, Mostafa M. Rezaee, Mehran Ghasemi, Robert C. Green

**Affiliations:** 1 Department of Computer Science, Bowling Green State University, Bowling Green, OH 43403 USA; 2 Department of Communication Sciences and Disorders, Bowling Green State University, Bowling Green, OH 43403 USA

**Keywords:** Generative artificial intelligence, Denoising diffusion probabilistic models, Dysphonia, Voice disorders, Vocal fold imaging, Vocal fold pathology

## Abstract

**Background:**

Voice disorders (VD) are often linked to vocal fold structural pathologies (VFSP). Laryngeal imaging plays a vital role in assessing VFSPs and VD in clinical and research settings, but challenges like scarce and imbalanced datasets can limit the generalizability of findings. Denoising Diffusion Probabilistic Models (DDPMs), a subtype of Generative AI, has gained attention for its ability to generate high-quality and realistic synthetic images to address these challenges.

**Purpose:**

This study explores the feasibility of improving VFSP image classification by generating synthetic images using DDPMs.

**Methods:**

404 laryngoscopic images depicting VF without and with VFSP were included. DDPMs were used to generate synthetic images to augment the original dataset. Two convolutional neural network architectures, VGG16 and ResNet50, were applied for model training. The models were initially trained only on the original dataset. Then, they were trained on the augmented datasets. Evaluation metrics were analyzed to assess the performance of the models for both binary classification (with/without VFSPs) and multi-class classification (seven specific VFSPs).

**Results:**

Realistic and high-quality synthetic images were generated for dataset augmentation. The model first failed to converge when trained only on the original dataset, but they successfully converged and achieved low loss and high accuracy when trained on the augmented datasets. The best performance was gained for both binary and multi-class classification when the models were trained on an augmented dataset.

**Conclusion:**

Generating realistic images of VFSP using DDPMs is feasible and can enhance the classification of VFSPs by an AI model and may support VD screening and diagnosis.

## Introduction

Voice (phonation) plays a vital role in speech production, perception, and thus communication. Voice is linked to the functioning of the vocal folds (VF). Consequently, any structural abnormalities of the VFs can disrupt their normal functioning and lead to voice disorders (VD). A VD is any deviation in pitch, loudness, or voice quality that can negatively impact communication and quality of life [1]. Around 17% of the general population may experience VDs at some point in their lives [2]. Furthermore, VD can impact a wide group of professionals, such as educators, singers, therapists, public speakers, and others [3–6]. Hence, individuals with VD often experience vocal discomfort and fatigue, alongside challenges in social interactions, professional performance, and well-being, which can diminish their overall quality of life [7–9]. Therefore, a timely assessment and accurate diagnosis of VD associated with vocal fold structural pathologies (VFSP) can lead to successful management.

The clinical evaluation of VD includes a multifaceted approach that involves a combination of subjective and objective assessments, including patient-reported surveys, auditory-perceptual assessments, acoustic evaluation, aerodynamic analysis, and laryngeal imaging procedures. Although these assessments are commonly used in many research and clinical settings, laryngeal imaging of the VF structure has become a crucial part of the clinical voice assessment, especially for evaluating VFSP. Hence, a remarkable number of professional, like otolaryngologists and speech-language pathologists (SLP), apply laryngeal imaging methods, such as laryngoscopy or stroboscopy, to examine VF structure in people with VD [10]. Moreover, laryngeal imaging is a crucial procedure that enables professionals to visualize the VF and improve diagnostic accuracy when evaluating VFSP, leading to more precise behavioral or surgical treatment [11].

However, several factors can pose challenges for examiners when using this procedure to evaluate VFSP. For example, differences in light and image transmission, along with varying image quality by utilized technique, can challenge examiners when applying these methods [12]. Furthermore, the examination of VF pathologies heavily relies on the examiner’s clinical experience, with trained experts able to provide more reliable diagnoses [13]. These challenges, thus, may affect the timely evaluation and accurate diagnosis of VFSP and related VD. Therefore, there is a critical need for further technological, methodological, and clinical advancement on laryngeal imaging to improve VD assessment and diagnosis. This progress can likely improve the accuracy of diagnosing VD and better support the application and effectiveness of voice treatments in daily communication [14].

In recent years, automated voice analysis has become increasingly utilized for the screening and diagnosis of VFSP. Accordingly, prominent advancements in technology have been developed for examining the larynx and obtaining different objective assessments of VD [15–17]. Although early efforts to develop systems for automated analysis of laryngeal images were limited, substantial progress has been made lately. Accordingly, Artificial Intelligence (AI) technology has gained special attention for its potential to address many of those discussed challenges by offering automated and objective analysis of VFSP [18]. AI has developed healthcare and revolutionized disease diagnosis, treatment, and patient well-being. AI systems apply constant algorithms to reduce variability in interpretation of medical imaging, enhancing diagnostic accuracy, and standardizing care across clinical settings [19]. Furthermore, AI can assist professionals in decision-making, addressing complex challenges, enhancing medical procedures, minimizing surgical invasiveness, enabling remote and telehealthcare, reducing healthcare costs, and delivering other benefits for the public good [20]. Moreove, technologies like AI are rapidly advancing and facilitating how various people needs are identified, assessed, and managed [21]. Hence, developing this technology is also rudimentary in enhancing healthcare services for individuals with VD.

Deep learning, a subtype of AI, has recently indicated a great potential in examining VFSP [22]. Hence, several studies have been conducted using a deep learning model to examine VD and VF structure. For example, Baldini et al. (2024) found that the deep learning models they used showed promise in identifying diagnostically relevant frames within laryngeal videos [23]. Lee et al. (2024) have applied such models to detect benign VFSP using connected speech and vowel sounds [24]. Although there have been many valuable contributions in this field, there are still some noticeable challenges.

First, AI research in VFSP has predominantly focused on detecting laryngeal cancer despite not being the only VFSP associated with VD. While laryngeal cancer can have severe effects on health and life quality, the prevalence of benign VFSP also requires attention. Conditions like Reinke’s edema, nodules, polyps, and cyst are more common than other VFSP and can be associated with VD, impacting life quality [25]. Moreover, considering more conditions for the classification process can enhance deep learning model accuracy and performance [26]. However, a critical challenge arises if the number of target classes increases. Expanding the number of classes complicates the classification process as it demands a larger dataset with a balanced distribution across all categories [27]. This challenge is further complex by the reliance of these studies on samples from participants with VFSP, which can restrict the dataset size and result in an imbalanced data distribution across classes.

**Fig. 1:**
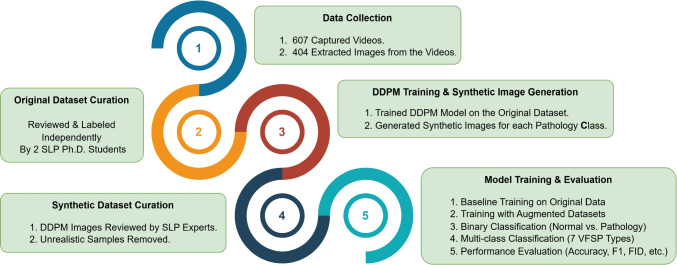
Workflow of the proposed study: from video-based data collection and expert-reviewed dataset curation to DDPM-based synthetic image generation and final model evaluation

Another challenge is that, in reality, the capability of different professionals to recruit or observe different VFSP may be influenced by factors like geographical location, area of expertise, available resources, The diverse epidemiology of VFSP, etc., which all may limit the generalizability of findings. This may eventually impact the quality of research, education, and, most importantly, patient outcomes. The effects may be even more prominent in the majority world, where most of the world’s population resides, and access to trained professionals, timely assessments, advanced technology, and accurate diagnoses can be limited [28]. Therefore, implementing more effective solutions may enhance the examination and detection of various VFSP using AI. To address these challenges, generative AI (GenAI) has gained special attention for its ability to use deep learning algorithms to generate synthetic data, such as realistic images, that closely resemble actual data. These synthetic data can supply more examples for training AI models without requiring additional patient data or when access to actual data is limited. Generating synthetic data, especially examples of underrepresented cases, help balance the dataset and improve model performance [29]. Hence, GenAI can expose AI models to a broader range of variations in pathological conditions, which can enhance their ability to generalize to real-life situations. As a result, GenAI has become a robust tool in improving healthcare services, disease diagnosis, and treatment options [30].

Within the last few years, Denoising Diffusion Probabilistic Models (DDPMs), a newer subtype of GenAI, have been developed and gained attention for their ability to produce high-quality realistic images [31]. DDPMs work by gradually adding noise to an image and then learning how to remove that noise. Once trained, they can generate realistic high-quality images that sets them apart from earlier models like Generative Adversarial Network (GAN) [32]. This capability allows DDPMs to produce images with superior quality, greater diversity, improved precision, and enhanced reliability compared to earlier models, which are all paramount in healthcare [33]. Moreover, DDPMs can handle scarce and imbalanced datasets better, where a dataset contains multiple classes but the distribution of data across these classes is imbalanced [34, 35]. These characteristics of DDPMs highlight their potential to address, at least to some extent, the limitations discussed earlier and to make the foundation for enhanced professional services for individuals with VF pathologies and VD.

Thus, this pilot study aims to investigate the feasibility of improving vocal fold structural pathology image classification by generating synthetic images using DDPMs. Our research questions are precisely outlined below:

**Primary research questions**

1. How feasible is generating images of vocal fold structural pathologies using the DDPM algorithm?
2. How is the preliminary outcome of classifying vocal fold structural pathology images using only the original dataset?
3. How feasible is it to enhance vocal fold structural pathology image classification using the original plus generated dataset?
4. What are the preliminary classification outcomes for binary classification (with and without pathology) compared to multi-class classification (seven specific pathologies)?

**Secondary research questions**

1. What are the key challenges in using DDPMs to improve classifying vocal fold structural pathology images?
2. What insights can be gained from this pilot study to design a larger-scale investigation into vocal fold structural pathology image classification using DDPMs?

To address the challenges outlined above, we designed a pilot study that integrates DDPM-generated synthetic images with deep learning classification models. The following section outlines the methods and experimental design used in this investigation.

**Table 1:** Data distribution

Label	Original data size	Synthetic data size
Without structural pathology	157	384
Nodule	71	374
Cyst	38	538
Polyp	35	585
Sulcus vocalis	33	315
Reinke’s edema	29	473
Keratosis	37	970
Granuloma	4	541
Total	404	4180

**Fig. 2:**
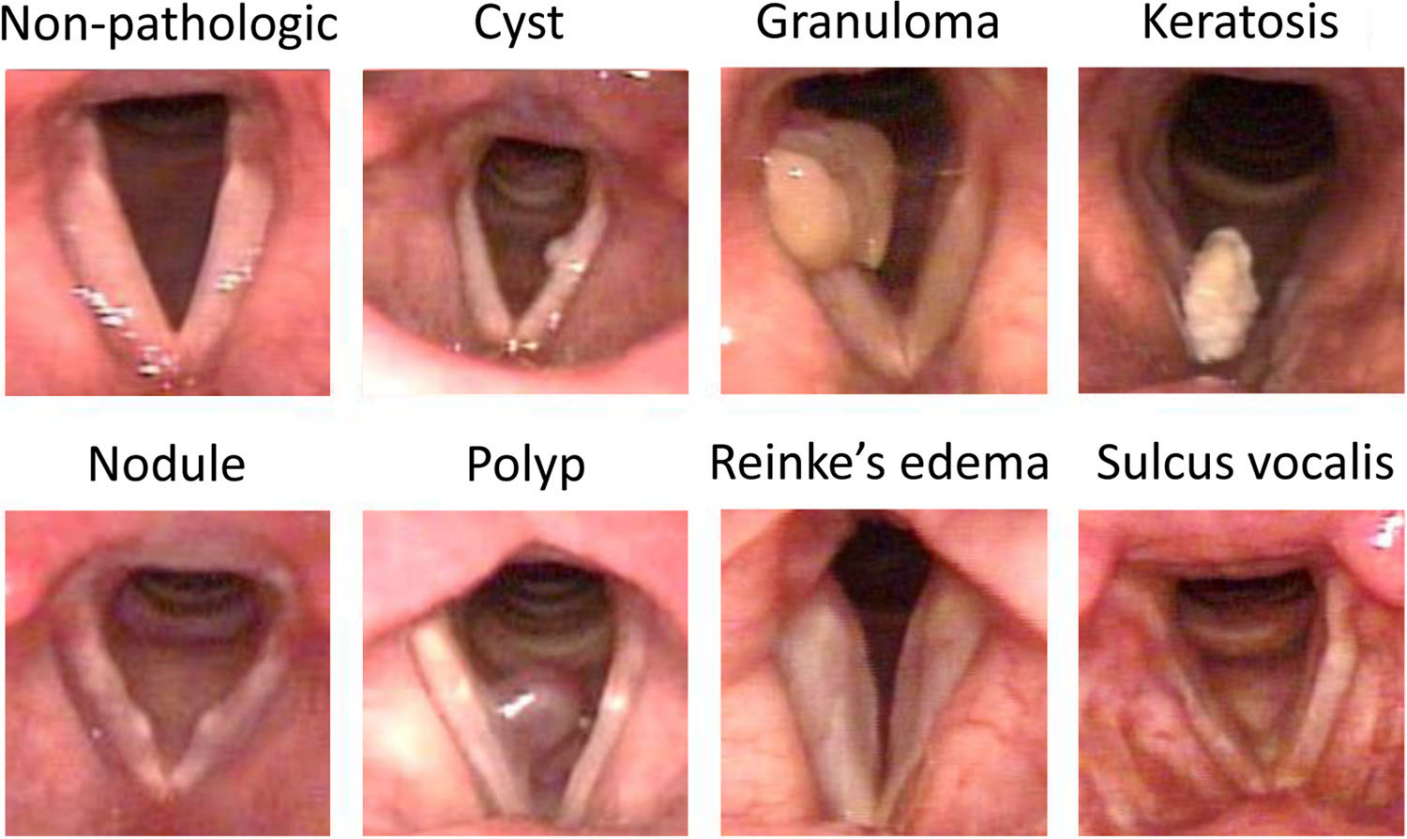
**Vocal Fold Structural pathologies.** Visual appearance of vocal fold structural pathologies selected for this study in addition to non-pathologic vocal folds (without observable pathology)

## Methods

This retrospective study was a voluntary collaboration between Ph.D. students in SLP and data analysis. Figure 1 presents an overview of the full experimental workflow, from data collection to model training and evaluation.

### Data collection

We utilized a collection of 607 de-identified and blinded laryngoscopic assessment videos provided by a colleague. All videos were recorded between 2014 and 2017 using the HighLight Basic Portable LED Stroboscope (INVENTIS s.r.l., Corso Stati Uniti, 1/3, 35127 Padova, Italy). All videos were saved in MPG format with dimensions of either 352 $\times$ 240 or 720 $\times$ 480 pixels and a frame rate of 30 frames per second.

### Original dataset curation

Two Ph.D. students in SLP, each with over five years of experience in laryngoscopic and stroboscopic evaluation and management of VD, independently reviewed the entire video dataset first. Then these expert SLPs examined and discussed the videos to familiarize themselves with the dataset. Next, they independently identified and labeled the types of pathologies depicted. The labeling process adhered to the descriptive criteria outlined in Atlas of Laryngoscopy by Robert et al. [36] and Classification Manual for Voice Disorders-I by Verdolini et al. [37] to ensure consistency and accuracy of evaluation. Moreover, unweighted Cohen’s Kappa ($\kappa$) was calculated using the irr and psych packages in *R* version (4.4.3) on Posit Cloud to measure the inter-rater reliability between the experts. Cohen’s Kappa is a commonly used coefficient for estimating inter-rater reliability between two raters. Accordingly, the analysis showed robust agreement ($\kappa$ = 0.86, SE = 0.02, 95% CI [0.83, 0.89], p < 0.001) between the two raters.

After the labeling process, we selected videos depicted VFSP, including no pathologies, nodules, polyps, cysts, Reinke’s edema, sulcus vocalis, granulomas, and keratoses for this study. Videos showing other types of pathologies were excluded. We also excluded videos that contained multiple VFSP or lacked sufficient clarity or duration for effective examination. After screening, a total of 404 videos were included in the original dataset (Table 1). The descriptions of each VF structural pathology included in this study are provided below [36, 37]. Figure 2 also shows visual appearance of these pathologies.

- **Nodule:** Nodules are benign, bilateral, and symmetrical VF mass. Nodules commonly appear near the midportion of the VFs, mostly where the anterior one-third of the VFs meets the posterior two-thirds.
- **Cyst:** A cyst is a benign encapsulated mass on the VFs, typically unilateral and near the midportion of the VFs. Cysts may cause reactive swelling or the formation of a mass on the contralateral side.
- **Polyp:** Polyps are benign focal deposits of gelatinous substance on the VFs, often associated with an enlarged blood vessel or hemorrhage. They commonly appear unilaterally and near the midportion of the VFs. They can be classified as pedunculated or sessile and may be either hemorrhagic or non-hemorrhagic, with different sizes.
- **Reinke’s edema:** Reinke’s edema is polypoid degeneration of the VFs. It is related to accumulation of gelatinous material in Reinke’s space, often bilateral, which can lead to swollen, floppy, or polypoid VFs.
- **Sulcus vocalis:** VF sulcus is a groove that extends partially or completely along the medial edge of one or both VFs. These grooves can be physiologic or anatomical.
- **Granuloma:** VF granuloma is a VF mass that is usually located near to the vocal processes (medial surface of the arytenoid), is typically unilateral, but can be bilateral.
- **Keratosis:** A keratotic lesion appears as a whitish patch that may be flat or raised, smooth or nodular, localized or diffuse, and surrounded by either normal or inflamed mucosa. Keratosis can mask a range of epithelial changes, from pre-malignant lesions such as leukoplakia to simple hyperplasia or invasive cancer like squamous carcinoma, serving as a surface-level indicator of an underlying pathological process. Early diagnosis of VF keratosis is crucial due to its potential for malignant transformation.

**Fig. 3:**
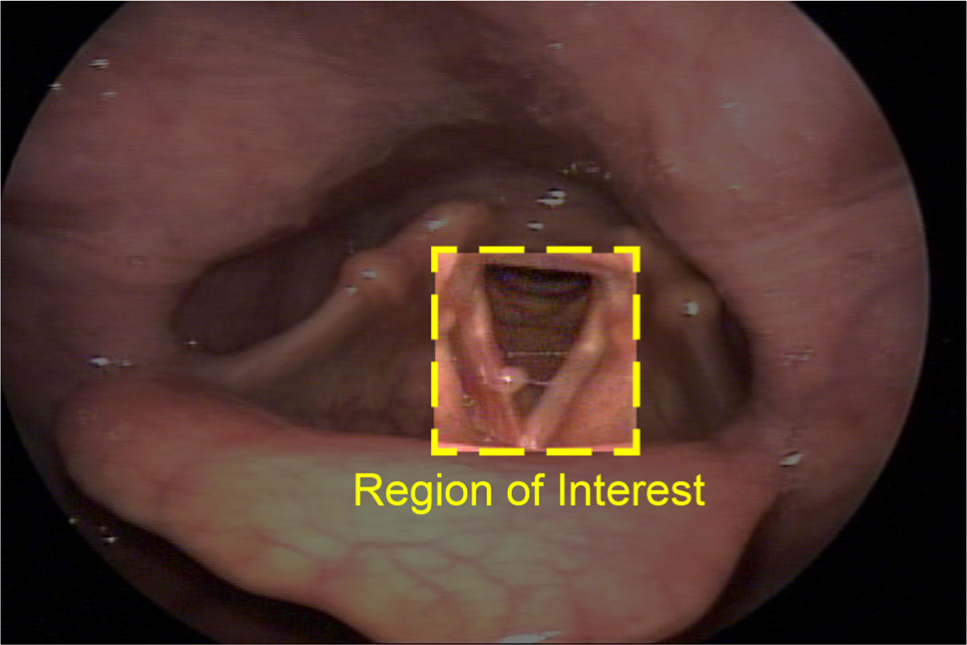
**Region of Interest**. Each selected image was manually cropped to a square shape, focusing on the region of interest (ROI). The use of ROI can prevent the processing of unnecessary image points and accelerate the process

Next, the videos were converted into images at a rate of 30 frames per second. Then the SLP experts selected the optimal frame from each video representing the identified pathology during VF abduction. Each selected image was then manually cropped to a square shape, focusing on the region of interest (ROI). The use of ROI can prevent the processing of unnecessary image points and accelerate the process (Fig. 3). As a result, the cropped images primarily focused on the abducted VFs and open glottis. All selected frames were saved in JPG format with dimensions of 240 $\times$ 240 pixels, a resolution of 300 dpi, and a 24-bit depth. These images made up the original dataset, which was the foundation for generating synthetic data.

The entire process of curating the original dataset took nearly four weeks.

### Synthetic dataset generation

This study utilized DDPM to generate synthetic images, augment the original dataset, and enhance classification performance. In this study, we utilized the Ohio Supercomputer Center (OSC) Pitzer Cluster, specifically the 48-core GPU-enabled nodes, each equipped with 2 NVIDIA Tesla V100 GPUs (32GB RAM per GPU) and 192GB of system RAM. The DDPM model was trained for 4 hours per experiment, and synthetic images were generated for each class within 3 hours. The training and inference processes were conducted using Python 3.8, PyTorch 1.13, CUDA 11.6, and cuDNN 8.4. These details have been added to enhance the clarity and reproducibility of our methodology. This process involved the following steps:

#### Forward diffusion process

The forward diffusion process starts with an original data sample $\textbf{x}_0$, and progressively adds Gaussian noise over $T$ timesteps. The corrupted image at time $t$, $\textbf{x}_t$, is defined as:

1 $$\begin{aligned} \textbf{x}_t = \sqrt{\bar{\alpha }_t} \textbf{x}_0 + \sqrt{1 - \bar{\alpha }_t} \varvec{\epsilon }, \end{aligned}$$

where:

- $\varvec{\epsilon } \sim \mathcal {N}(0, \textbf{I})$ is Gaussian noise.
- $\bar{\alpha }_t$ is a variance schedule controlling the level of noise added at each timestep.

The variance schedule $\bar{\alpha }_t$ is computed using a cosine function to ensure a smooth noise transition:

2 $$\begin{aligned} \bar{\alpha }_t = \frac{f(t)}{f(0)}, \quad f(t) = \cos ^2\left( \frac{t / T + s}{1 + s} \cdot \frac{\pi }{2}\right) , \end{aligned}$$

where $s$ is a small constant offset to avoid very small noise levels.

This process transforms the data distribution into a Gaussian noise distribution over $T$ timesteps.

#### Reverse diffusion process

The reverse process learns to denoise $\textbf{x}_t$ back to $\textbf{x}_0$. The model predicts the noise $\varvec{\epsilon }_\theta$ at each timestep and iteratively refines the data. The reverse process is parameterized as:

3 $$\begin{aligned} p_\theta (\textbf{x}_{t-1} | \textbf{x}_t) = \mathcal {N}\left( \textbf{x}_{t-1}; \varvec{\mu }_\theta (\textbf{x}_t, t), \sigma _t^2 \textbf{I}\right) , \end{aligned}$$

where $\varvec{\mu }_\theta$ is the predicted mean, and $\sigma _t^2$ is the variance at timestep $t$. The model is trained to minimize the reconstruction loss:

4 $$\begin{aligned} L = \mathbb {E}_{\textbf{x}_0, \varvec{\epsilon }, t}\left[ \Vert \varvec{\epsilon } - \varvec{\epsilon }_\theta (\textbf{x}_t, t)\Vert ^2\right] . \end{aligned}$$

Here, $\varvec{\epsilon }_\theta (\textbf{x}_t, t)$ represents the model’s prediction of the noise added at timestep $t$.

#### Architecture

The DDPM uses a U-Net architecture for the reverse process. Key components include:

- **Sinusoidal positional embeddings:** Encoding timestep $t$ to inform the model about the noise level in the input.
- **Wide ResNet blocks:** Including convolutional layers, normalization, and SiLU activations to process image features effectively.
- **Skip connections:** Preserving fine-grained details by combining features from different levels of the U-Net.

#### Training and image generation

- **Training phase:** The forward process corrupts images using the noise schedule. The model is then trained to predict the added noise at each timestep by minimizing the reconstruction loss. Training the model was conducted using the following hyperparameter configurations.
  - **Epochs:** 1,024
  - **Steps:** 10,000
  - **Learning rate:** $$ 1 \times 10^{-4} $$
  - **Input size:** $$ 256 \times 256 $$
  - **Batch size:** 32
- **Image generation phase:** Starting with pure noise, the reverse process iteratively removes noise and reconstruct a realistic image.

The DDPM architecture used in this study is based on a U-Net structure (see Step by Step Visual Introduc-tion to Diffusion Models). This architecture can integrate essential components, such as skip connections, attention mechanisms, and normalization techniques, to ensure stable training and high-quality image generation [38]. The U-Net structure can connect the downsample and upsample paths, allowing for the preservation and refinement of critical details throughout the reverse diffusion process. The training and image generation processes were performed on the Ohio Supercomputer Center’s Pitzer Cluster using 48-core GPU-enabled nodes equipped with two NVIDIA Tesla V100 GPUs (32 GB each). DDPM training required approximately 4 hours, and image generation for each class took about 3 hours. CNN models were trained and evaluated in under 30 minutes per run. While detailed profiling was not performed, these runtime estimates reflect the general computational efficiency of our workflow. By employing this design, the DDPM used in this study can generate diverse and realistic synthetic images, addressing challenges such as spatial consistency and feature coherence, which are crucial for medical imaging applications like the classification of VFSP.

### Synthetic dataset curation

**Fig. 4:**
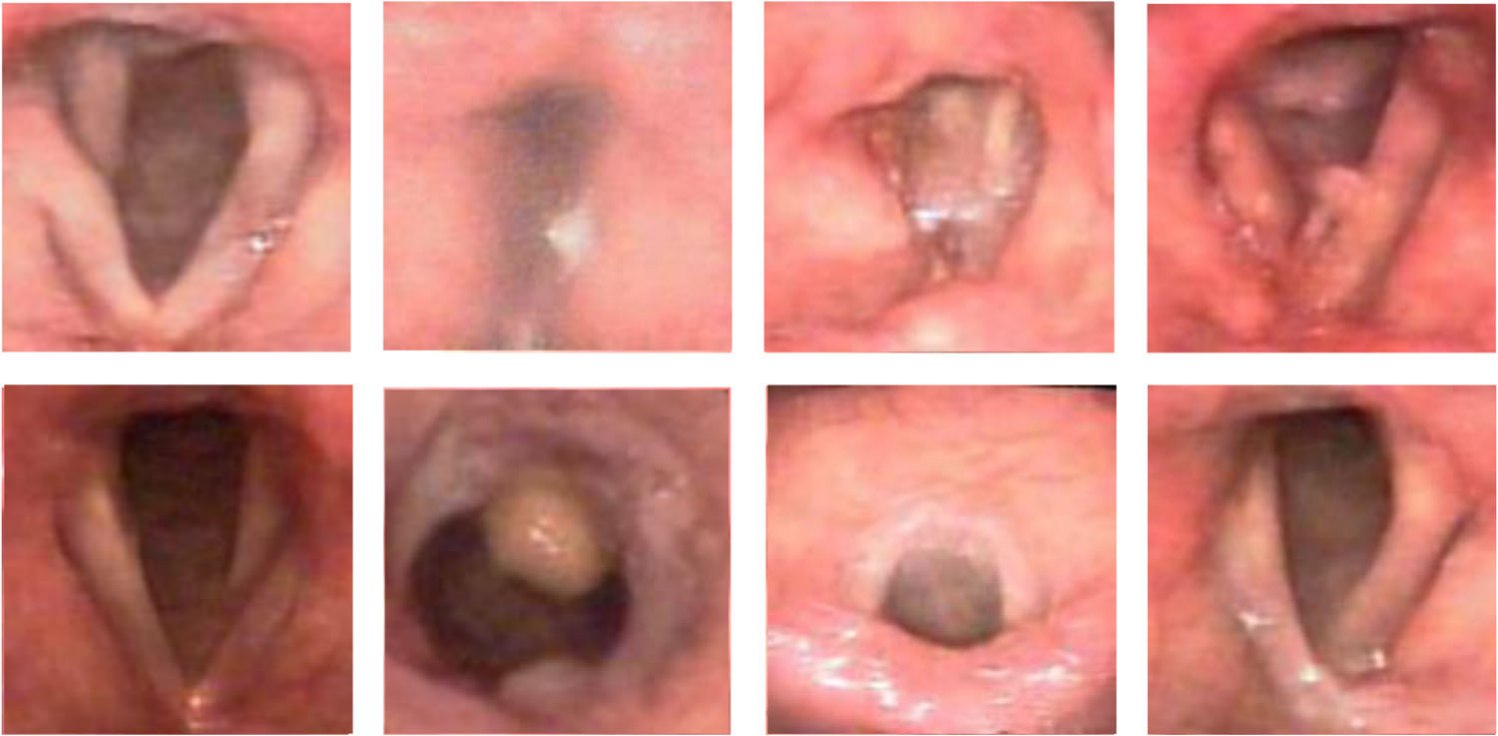
**Unrealistic Synthetic Images Removed by the SLP Experts**. These images, generated using DDPM, were deemed structurally inaccurate or unrepresentative of real pathologies during the evaluation process

Synthetic images generated by the DDPM were reviewed by the SLP experts to ensure clinical relevance. Accordingly, they reconsidered the descriptive criteria outlined in Atlas of Laryngoscopy by Robert et al. and Classification Manual for Voice Disorders-I by Verdolini et al. to evaluate the synthetic data [36, 37]. Moreover, the next primary criteria were to identify and discard any deformed or unrealistic examples. For example, some synthetic images failed to capture the realistic configuration of the VFs, leading to instances where three VFs appeared or the glottis was unidentifiable. A further example was when no VFs appeared in the image. Another criterion was to exclude any example that was remarkably blurry. Figure 4 shows such examples. This curation process was performed to ensure that only high-quality and realistic synthetic images were included in the augmented dataset.

### Model training and evaluation

In this study, we applied two well-established convolutional neural network (CNN) architectures, VGG16 and ResNet50, for model training and evaluation.

VGG16 is known for its simplicity and uniform structure, comprising different layers that include convolutional, pooling, and fully connected layers [39]. This architecture excels in feature extraction due to its deep and sequential design, making it highly effective for image classification tasks. Accordingly, we first used untrained VGG16, which starts with random weights, having no prior knowledge of features. This setup allows the model to learn directly and solely from the dataset. We also used pre-trained VGG16. Pre-trained models are usually trained on larger datasets, which enables them to learn generic visual features such as edges, textures, and object shapes. Accordingly, they have prior knowledge of features. Using pre-trained models leverages prior knowledge and makes it useful for transfer learning when applying to small or specialized datasets like our dataset. Thus we applied this to explore how a model with pre-learned features performs relative to the other.

During loading, images were converted to arrays and normalized using the VGG16 preprocessing function to ensure compatibility with pretrained models. For CNN training, we used a batch size of 16, a learning rate of 1e-4 with the Adam optimizer, and the sparse categorical crossentropy loss function. The dataset was split using stratified sampling (80% training, 20% validation), and each model was trained for 20 epochs. No further hyperparameter tuning was performed for the CNNs to maintain consistency.

For DDPM training, we performed a manual grid search over multiple configurations. We experimented with different numbers of epochs (256, 512, 1024, 2048, and 3072) and timesteps (1000, 3000, 5000, 8000, and 10000). After evaluating the realism and diversity of the generated images, we selected the optimal settings: 3072 epochs, 10000 timesteps, batch size of 16, image size of 128$\times$128, mixed precision (fp16), and a gradient accumulation step of 1.

On the other hand, ResNet50 employs residual learning through skip connections, which addresses the vanishing gradient problem in deeper networks [40]. With various layers, ResNet50 provides a more complex feature representation, enabling the model to capture intricate patterns within the data. We accordingly used pre-trained ResNet50 as it benefits from prior training on larger generic datasets. We used ResNet50 to compare its performance against VGG16, which can indicate whether a deeper, more sophisticated model adds value to the classification task.

#### Baseline model training

Models were first trained exclusively on the original dataset to establish baseline performance. This phase was conducted to explore the effectiveness of incorporating synthetic data in subsequent experiments.

#### Binary classification using synthetic data

Binary classification experiments were conducted to evaluate models’ ability to differentiate between vocal folds *without* and *with structural pathology.* The experiments were conducted under three distinct training scenarios to explore the impact of synthetic data and its combination with original data on classification tasks. Hence, the models were first trained only on the synthetic dataset generated by DDPM and tested on the entire original dataset. Next, the models were trained on a dataset that included synthetic images augmented with 10% of the original data, while the remaining 90% of the original data was used for testing. Third, the models were trained on a dataset that included synthetic images augmented with 50% of the original data, with the remaining 50% of the original dataset reserved for testing.

#### Multi-class classification using snthetic data

For multi-class classification, models were trained to classify those seven types of VFSP (i.e., *Cyst, Granuloma, Keratosis, Nodule, Polyp, Reinke’s Edema, and Sulcus Vocalis*). The experiments here were also conducted under the three similar distinct training scenarios mentioned above.

#### Metrics for evaluation

To assess the performance of the classification models, we employed standard evaluation metrics, including accuracy, F1 Score, sensitivity (recall), specificity, precision (positive predictive value), and Fréchet Inception Distance, which are commonly used in medical imaging. Moreover, confusion matrix were created for each model.

##### Accuracy

Accuracy represents the proportion of correctly classified samples out of the total samples. A higher accuracy can indicate the overall ability of the model to correctly identify both VF imaging with (true positive (TP)) and without (true negative (TN)) pathologies but does not differentiate between the types of errors made (e.g., false negatives (FN) from false positives (FP)).

5 $$\begin{aligned} \text {Accuracy} = \frac{\text {TP} + \text {TN}}{\text {TP} + \text {FP} + \text {FN} + \text {TN}} \end{aligned}$$

##### Precision (Positive Predictive Value)

Precision measures the proportion of true positive diagnoses among all positive predictions. A higher precision reflects that the majority of predicted positive cases (e.g., a specific type of structural pathology) are accurate, effectively reducing the occurrence of false positives.

6 $$\begin{aligned} \text {Precision} = \frac{\text {TP}}{\text {TP} + \text {FP}} \end{aligned}$$

##### Sensitivity (recall)

Sensitivity is the ability of the model to correctly identify true positive cases. Sensitivity reflects the model’s capacity to detect the pathologic condition when they are present. A higher sensitivity can ensure that no pathologic cases are missed.

7 $$\begin{aligned} \text {Sensitivity} = \frac{\text {TP}}{\text {TP} + \text {FN}} \end{aligned}$$

##### F1 score

The F1 score is the harmonic mean of precision and sensitivity, offering a balanced measure that accounts for both false positives and false negatives. The F1 score is particularly valuable when dealing with imbalanced datasets, where certain classes have fewer samples. The F1 score ensures that the model performs well on both detecting lesions and avoiding over-prediction. Indeed, a higher F1 score indicates that a model is able to accurately identify positive cases while minimizing false positives and false negatives.

8 $$\begin{aligned} F1 \, \text {Score} = 2 \cdot \frac{\text {Precision} \cdot \text {Sensitivity}}{\text {Precision} + \text {Sensitivity}} \end{aligned}$$

##### Specificity

Specificity measures the proportion of true negatives correctly identified among all negative cases. Specificity indicates the model’s ability to correctly identify people without pathological condition. A higher specificity can ensure that fewer non-pathologic cases are missed.

9 $$\begin{aligned} \text {Specificity} = \frac{\text {TN}}{\text {TN} + \text {FP}} \end{aligned}$$

##### Fréchet Inception Distance

Fréchet Inception Distance (FID) evaluates the similarity between the synthetic images generated by DDPM and the original images. A lower FID score represents that the synthetic images closely resemble the actual ones in terms of visual and anatomical appearance.

10 $$\begin{aligned} \text {FID} = ||\mu _r - \mu _s||^2 + \text {Tr}(\Sigma _r + \Sigma _s - 2(\Sigma _r \Sigma _s)^{1/2}) \end{aligned}$$

where $\mu _r, \mu _s$ are the means and $\Sigma _r, \Sigma _s$ are the covariances of the features extracted from real and synthetic images.

##### Confusion matrix

A confusion matrix, also known as error matrix, is a chart that provides a clear overview of a classification algorithm’s performance. It serves as a tool to visualize and summarize how well the algorithm classifies data by showing the actual and predicted class labels.

The experimental procedures described above were applied to both original and synthetic datasets. The next section presents the outcomes of these experiments and evaluates the models’ performance.

## Results

### Original dataset

The present study included 404 original images, which exhibited imbalance in VFSP distribution (Table1). The *Granuloma* class had the fewest samples, with just 4 instances, whereas the *without pathology* class was the most represented, comprising 157 samples, which was nearly 40 times bigger than the least frequent class size.

### Synthetic dataset

**Fig. 5:**
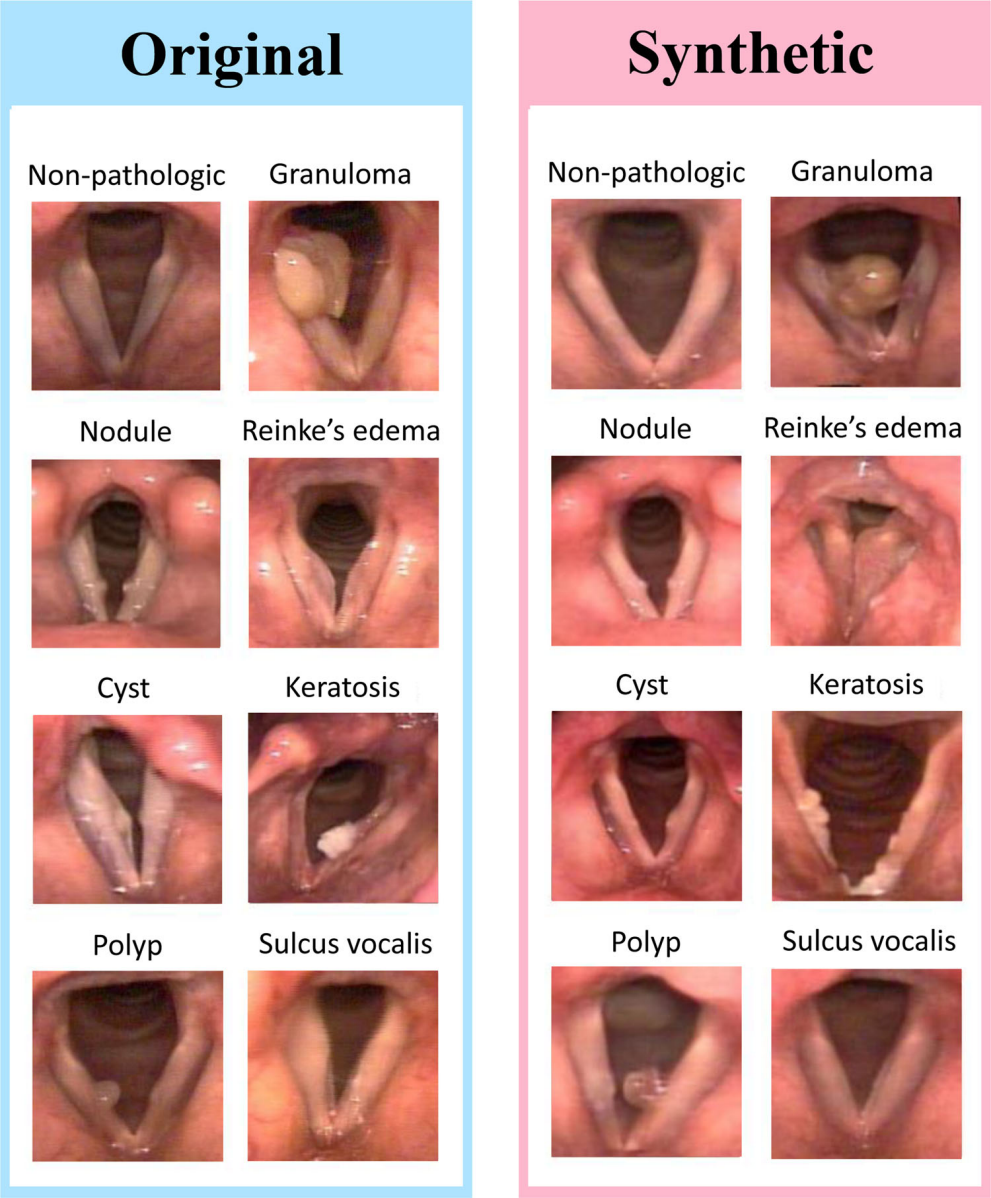
**Original and synthetic images**. This figure shows examples of original images and those generated by DDPM

The synthetic dataset was built and reviewed by the SLP experts. After they excluded any deformed or unrealistic synthetic images (e.g., inaccurate VF structure; see Fig. 4), the included generated dataset comprised 4180 synthetic realistic images (Table 1), which closely resembled original images in terms of anatomical appearance and fidelity (Fig. 5). Hence, the dataset scarcity and imbalance was addressed.

**Fig. 6:**
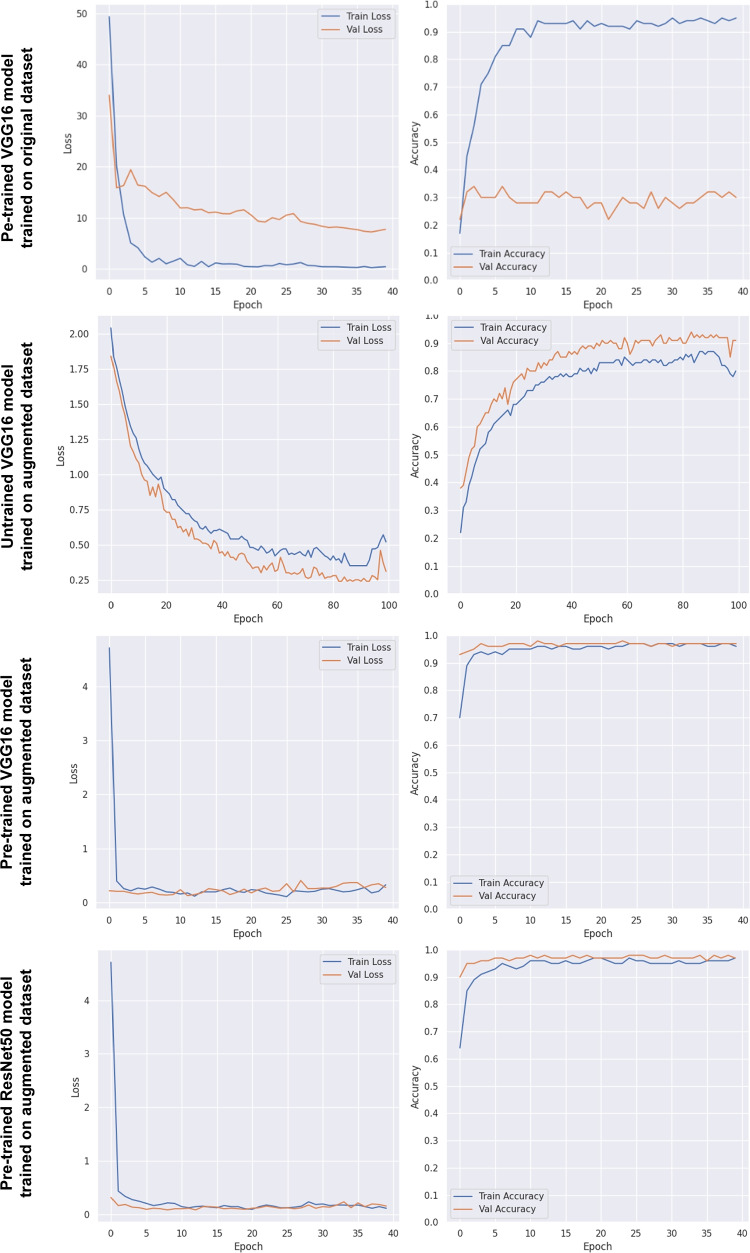
**Training and Evaluation**. The training and validation loss and accuracy were compared across models trained on the *original dataset* and the *augmented dataset*. Except for the pre-trained VGG16 model trained only on the original dataset, all other models achieved convergence when the dataset was augmented with synthetic images

**Table 2:** Evaluation metrics across all binary classification experiments

	Trained on synthetic data	Mixed with 10% original data	Mixed with 50% original data
Pre-trained VGG16 results			
Test Accuracy	0.70 ± 0.03	0.70 ± 0.02	0.71 ± 0.03*
F1 Score	0.66 ± 0.03	0.64 ± 0.03	0.68 ± 0.03*
Sensitivity	0.70 ± 0.03	0.69 ± 0.03	0.71 ± 0.03*
Specificity	0.29 ± 0.09*	0.25 ± 0.09	0.28 ± 0.10
Precision	0.76 ± 0.02	0.77 ± 0.02*	0.77 ± 0.02*
Pre-trained ResNet50 results			
Test Accuracy	0.70 ± 0.03	0.72 ± 0.03	0.73 ± 0.03*
F1 Score	0.64 ± 0.03	0.69 ± 0.03	0.70 ± 0.03*
Sensitivity	0.69 ± 0.03	0.71 ± 0.03	0.73 ± 0.03*
Specificity	0.23 ± 0.05	0.28 ± 0.12	0.31 ± 0.12*
Precision	0.75 ± 0.03	0.77 ± 0.02	0.78 ± 0.03*
Untrained VGG16 results			
Test Accuracy	0.69 ± 0.04	0.67 ± 0.03	0.74 ± 0.04*
F1 Score	0.63 ± 0.03	0.60 ± 0.03	0.69 ± 0.04*
Sensitivity	0.69 ± 0.03	0.68 ± 0.03	0.74 ± 0.04*
Specificity	0.21 ± 0.07	0.14 ± 0.05	0.40 ± 0.03*
Precision	0.73 ± 0.03	0.73 ± 0.03	0.78 ± 0.03*

*Note.* (*) shows the highest evaluation metric among the others. The left column shows the results when the models were trained solely on the synthetic dataset. The middle column indicates the results when the models were trained on a dataset that included synthetic images augmented with 10% of the original data, while the remaining 90% of the original data was used for testing. The right column displays the results when the models were trained on a dataset that included synthetic images augmented with 50% of the original data, with the remaining 50% of the original dataset reserved for testing

**Table 3:** Evaluation metrics across all multi-class classification experiments

	Trained on synthetic data	Mixed with 10% original data	Mixed with 50% original data
Pre-trained VGG16 results			
Test Accuracy	0.79 ± 0.03	0.82 ± 0.03	0.83 ± 0.02*
F1 Score	0.78 ± 0.04	0.82 ± 0.04	0.82 ± 0.03*
Sensitivity	0.79 ± 0.03	0.82 ± 0.03	0.83 ± 0.02*
Specificity	0.96 ± 0.01	0.97 ± 0.01	0.97 ± 0.01*
Precision	0.81 ± 0.03	0.84 ± 0.03	0.85 ± 0.02*
Pre-trained ResNet50 results			
Test Accuracy	0.78 ± 0.03	0.78 ± 0.03	0.82 ± 0.02*
F1 Score	0.77 ± 0.04	0.77 ± 0.04	0.81 ± 0.03*
Sensitivity	0.78 ± 0.03	0.78 ± 0.03	0.82 ± 0.02*
Specificity	0.96 ± 0.01	0.96 ± 0.01	0.97 ± 0.01*
Precision	0.80 ± 0.02	0.80 ± 0.02	0.84 ± 0.02*
Untrained VGG16 results			
Test Accuracy	0.71 ± 0.04	0.71 ± 0.03	0.74 ± 0.05*
F1 Score	0.70 ± 0.04	0.71 ± 0.04	0.73 ± 0.04*
Sensitivity	0.70 ± 0.04	0.71 ± 0.03	0.74 ± 0.05*
Specificity	0.95 ± 0.01	0.94 ± 0.01	0.96 ± 0.01*
Precision	0.73 ± 0.04	0.75 ± 0.03	0.78 ± 0.03*

*Note.* Each (*) shows the highest evaluation metric among the others. The left column shows the results when the models were trained only on the synthetic dataset. The middle column indicates the results when the models were trained on a dataset that included synthetic images augmented with 10% of the original data, while the remaining 90% of the original data was used for testing. The right column displays the results when the models were trained on a dataset that included synthetic images augmented with 50% of the original data, with the remaining 50% of the original dataset reserved for testing

### Model training and evaluation

#### Baseline model training

The training and validation loss and accuracy for the pre-trained VGG16 model trained exclusively on the original dataset can be found in Fig. 6. The training process revealed that the model failed to converge, with the validation accuracy remaining consistently low throughout the epochs.

#### Binary classification using synthetic data

The training process using the synthetic data demonstrated that all models effectively converged (Fig. 6). Accordingly, all models achieved low loss and high accuracy during training and validation. Pre-trained VGG16 and ResNet50 consistently exhibited better performance and faster convergence compared to the untrained VGG16.

Table 2 provides a summary of the evaluation metrics for binary classification across all experiments. Higher evaluation metrics were achieved when the models were trained on datasets augmented with synthetic images and 50% of the original data, while the remaining 50% was reserved for testing. Lower metrics were gained when the models were trained only on synthetic datasets.

#### Multi-class classification of structural pathologies

For multi-classification, the training process using synthetic data demonstrated that all models successfully converged. Across all models, training exclusively on synthetic data yielded the lowest performance. Adding a small portion (10%) of original data improved performance across all models. Finally, combining synthetic data with a larger portion (50%) of the original dataset led to the highest performance in all models.

Table 3 provides a summary of the evaluation metrics for multi-class classification across all experiments. Better evaluation metrics were obtained when the models were trained on datasets that combined synthetic images with 50% of the original data, with the remaining 50% set aside for testing.

The calculated FID metric presented in Table 4. The FID metric demonstrated variability in similarity across pathology classes. The FID scores, from lower to higher, were found in nodule, cyst, polyp, keratosis, Reinke’s edema, granuloma, and sulcus vocalis categories, respectively.

**Fig. 7:**
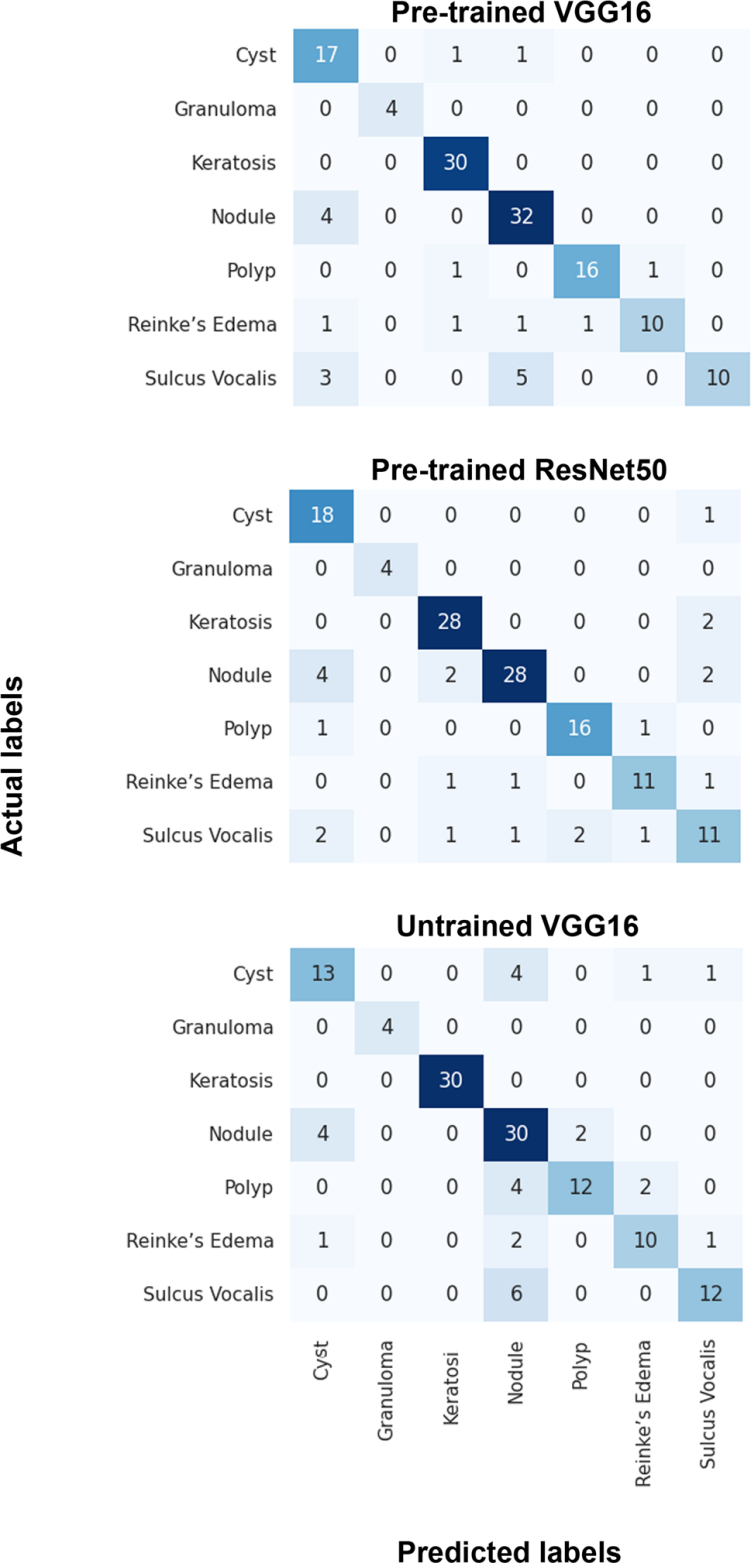
**Confusion Matrix**. These confusion matrices show the performance of the three models trained on a dataset that included synthetic images augmented with 50% of the original data, while the remaining 50% of the original dataset kept for testing

Finally, Fig. 7 displays the confusion matrices for the top-performing models. As previously mentioned, these models achieved the best results when trained on datasets combining synthetic images with 50% of the original data, while the remaining 50% was reserved for testing. Accordingly, the pre-trained VGG16 showed the most robustness in correctly classifying granulomas and keratoses. Although it did pretty well on classifying other categories, it occasionally misclassified nodules as cysts and sulcus vocalis as either nodules or cysts. The pre-trained ResNet50 did better on classifying nodules, keratoses, granuloma, and cysts, while sometimes misclassified nodules as cysts. Eventually, the untrained VGG16 indicated the most strength in accurately classifying nodules, keratoses, and granuloma, but occasionally misclassified sulcus vocalis, polyps, and cysts as nodules.

The results highlight the effectiveness of using synthetic images to enhance model convergence and classification performance. These findings are further interpreted and contextualized in the following discussion.

## Discussion

To the best of our knowledge, this study explored the feasibility of enhancing the classification of VFSP images by generating synthetic images using DDPM for the first time. Accordingly, we utilized two well-established convolutional neural network (CNN) architectures, VGG16 and ResNet50, for model training and evaluation. Initially, the models were trained only on the original dataset to establish the baseline performance. We then conducted binary classification experiments to evaluate the models’ ability to distinguish between vocal folds *without* and *with* structural pathology. Furthermore, we trained the models to classify seven specific types of VFSP, including *cyst, granuloma, keratosis, nodule, polyp, Reinke’s edema, and sulcus vocalis*. The findings of the study were promising and illustrated potential improvements in classification performance.

**Table 4:** Frechet Inception Distance (FID) scores

Label	FID Score
Nodule	104.70
Cyst	112.00
Polyp	120.70
Keratosis	140.64
Reinke’s Edema	163.94
Granuloma	226.45
Sulcus Vocalis	227.31

*Note.* FID scores were calculated for individual VF structural pathology categories. Lower FID score shows more similarities between original and synthetic images

The synthetic dataset generated by DDPM effectively addressed the scarcity and imbalance in our original dataset. It closely resembled the actual dataset in terms of anatomical accuracy and fidelity, which highlights its potential for augmenting laryngoscopic imaging datasets. This observation aligns with Deshpande et al., who demonstrated that DDPMs achieve lower contextual error rates in synthetic medical images [41]. Furthermore, DDPM’s ability to produce images with enhanced diversity and fewer artifacts has been highlighted in prior studies [42]. Consistent with these findings, our study revealed that DDPM is capable of generating high-quality, diverse, and realistic laryngoscopic images with minimal artifacts.

After generating the synthetic dataset, we used both untrained and pre-trained models for training and evaluation. Preliminary findings revealed that the model failed to converge when trained exclusively on the original dataset. However, augmenting the original dataset even with a small portion of synthetic images during training enabled the models to successfully converge. The initial poor performance can highlight the limitations posed by the small and imbalanced nature of the original dataset, which likely lacks sufficient diversity and representation across classes. These findings can imply that training a model only on the original laryngoscopic dataset might not be sufficient to achieve reliable classification results. Therefore, augmenting a scarce real dataset with synthetic images can enable deep learning models to successfully converge [43]. This convergence occurred more quickly and sharply when pre-trained models were used relative to the untrained model (Fig. 6). This highlights the notion that fine-tuning pre-trained models can achieve faster convergence during training compared to using untrained models, even when working with laryngoscopic images. Therefore, our findings suggest that augmenting a laryngoscopic dataset with synthetic images, along with fine-tuning pre-trained models, can lead to faster and more successful model convergence.

Evaluation metrics for both binary (*with* and *without* VFSP) and multi-class classification (seven specific VFSPs) revealed that the best performance was achieved when the models were trained on a dataset augmented with a larger proportion of synthetic images. This suggests that augmenting the laryngoscopic dataset with a larger proportion of synthetic images can enhance the performance. For binary classification, untrained and pre-trained models both showed remarkable results. All metrics, except for specificity, were acceptable, suggesting that these models have the potential to be developed for screening people with VFSP. Indeed, tools with higher sensitivity are more capable of screening as they rarely miss subjects with pathological conditions [44]. For multi-class classification, untrained and pre-trained models both indicated promising results. All metrics were acceptable, suggesting that these models have the potential to be developed for screening as well as diagnosing specific VFSP. Contrarily to the binary classification, all models demonstrated robust specificity, highlighting their potential to be developed into a diagnostic tool. Indeed, tools with higher specificity are more efficient for diagnostic purposes as they rarely miss subjects without pathological conditions [44]. Finally, the FID metric showed variability in the similarity across different classes, which might be due to dissimilarity in the visual appearance of different VFSPs, as these pathologies may occur in distinct subtypes or configurations.

We also encountered two challenges during this study that should be considered for future research. First, we lacked access to a larger or higher-quality dataset, such as high-speed laryngoscopic videos, which could have provided more insights into how sample size or image quality might influence these preliminary findings. The lack of publicly available laryngoscopic datasets might limit external validation and generalizability. Future work will seek collaborations to obtain external datasets for validation and broader applicability. Additionally, due to limited resources, we did not have access to an otolaryngologist, whose expertise could have enhanced the research process by validating the data. By addressing these limitations, future research can further expand our understanding of using DDPMs to improve VFSP image classification through synthetic image generation. Moreover, future research should focus on a similar investigation based on histological diagnoses rather than relying only on visual manifestations. In addition, future efforts can focus on refining and expanding the capabilities of the model, such as enabling it to indicate the anatomical side and location of the VFSP. Future research can also use diversity indices, such as the Shannon Diversity Index [45], to evaluate how generative models impact the diversity of laryngoscopic datasets.

In summary, this study provides valuable insights into the use of generative models for VFSP classification. The concluding section synthesizes these findings and outlines their potential implications for clinical practice and future research.

## Conclusion

Voice disorders can commonly lead to communication issues that can affect social interaction and life quality. Structural pathologies of the vocal folds can disrupt their normal functioning, leading to voice disorders. This study demonstrated that generating images of vocal fold structural pathologies using DDPMs is feasible and holds potential for enhancing the classification of these pathologies and supporting voice disorder screening or diagnosis. While AI is not replacement for human professionals, these models have the potential to support practitioners and improve healthcare services for individuals with voice disorders worldwide. Therefore, future studies should explore this capability on a larger scale.

## Data Availability

The authors verify that the data supporting the findings of this study are available within the article. Researchers interested in studying our dataset or collaborating are encouraged to contact the authors or refer to this GitHub link: https://github.com/imankhazrak/DDPM_Laryngeal-Lesions.
